# Simultaneous Osseo- and Odontointegration of Titanium Implants: Description of Two Cases in Human and Animal Models and Review of Their Experimental and Clinical Implications

**DOI:** 10.3390/ma17225555

**Published:** 2024-11-14

**Authors:** Iván Valdivia-Gandur, María Cristina Manzanares-Céspedes, Wilson Astudillo-Rozas, Oscar Aceituno-Antezana, Victòria Tallón-Walton, Víctor Beltrán

**Affiliations:** 1 Biomedical Department, Universidad de Antofagasta, Avenida Angamos 601, Antofagasta 1270300, Chile; ivan.valdivia@uantof.cl (I.V.-G.); wastudro11@alumnes.ub.edu (W.A.-R.); oscar.aceituno@uantof.cl (O.A.-A.); 2 Human Anatomy and Embryology Unit, Universitat de Barcelona, 08193 Barcelona, Spain; vtallon@ub.edu; 3 Dentistry Department, Universidad de Antofagasta, Avenida Angamos 601, Antofagasta 1270300, Chile; 4 Clinical Investigation and Dental Innovation Center (CIDIC), Dental School and Center for Translational Medicine (CEMT-BIOREN), Universidad de La Frontera, Temuco 4811230, Chile

**Keywords:** titanium implant, osseointegration, cementoconduction, cementointegration, dental tissue engineering, biomaterial

## Abstract

Two cases of calcified bone and dental tissue integration with titanium implants are presented, along with a review of the literature on their experimental and clinical implications. First, histological analyses of a titanium implant extracted from a patient with iimplant disease revealed the integration of both dental and bone tissue on the implant’s surface. Secondly, a biocompatibility study in an animal model documented two implants in contact with tooth roots. Samples from both animal and human models demonstrated simultaneous osseointegration and dental tissue neoformation, with the latter attributed to the activity of cementoblasts. The literature review confirms the formation of cementum around dental implants in contact with teeth. Certain clinical reports have proposed the insertion of implants into bone sites containing impacted teeth as a conservative treatment alternative, avoiding the need for tooth extraction surgery and demonstrating the successful integration of teeth, bone, and dental implants. Furthermore, the documented natural formation of periodontal tissues around dental implants provided a foundation for tissue engineering studies aimed at realizing implant–bone relationships similar to those of natural bone–tooth structures. The primary challenges remain the long-term preservation of periodontal-like tissue formed on implants and the imparting of functional proprioceptive properties.

## 1. Introduction

The processes of osseointegration, osseoinduction, and osteoconduction have been extensively studied [1,2]. However, knowledge regarding the behavior of dental and periodontal tissues in contact with dental implants remains limited. Currently, at least three major points of discussion are associated with this phenomenon. The first is the incorporation of dental tissue within the mandibular or maxillary bone as part of the implant bed. The second is the application of tissue engineering to realize implant integration with bone tissue through periodontal-like tissue. The third is the preservation of a layer of dental tissue for alveolar ridge maintenance during implant bed preparation; this special layer is subsequently maintained in contact with the dental implant. These topics are of significant interest from histological, functional, and dental tissue engineering perspectives. The aim of this study is to report two cases of dental implant integration via dental, periodontal, and bone tissues in both a human and an animal model and to review the literature on the clinical and experimental implications of this phenomenon in the discussion.

## 2. Case Descriptions

### 2.1. Findings in Human Sample

**Figure 1:**
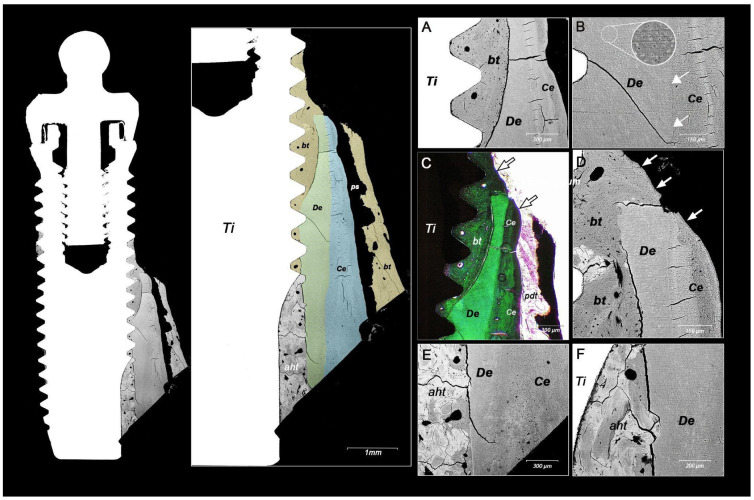
Dental tissue in contact with a dental implant in a human sample obtained after implant extraction due to peri-implant disease. The two images on the left display the complete sample, with dental tissue and bone in contact with the implant surface (Ti). Bone tissue (bt) is highlighted in yellow, dentin (De) in green, and cementum (Ce) in light blue. The periodontal space (ps) is visible near the dental tissue (cementum). The lower portion of the image shows an amorphous hard tissue (aht) containing bone tissue fragments, apparently integrated with newly formed cementum between the dentin and implant surface. Images (**A**–**F**) present histological details from the sample. In (**A**), bone tissue is observed between the implant threads and dental tissue (dentin and cementum). In (**B**), perforations on the surface indicate dentinal tubules (enhanced in the white circle with increased contrast); additionally, the boundary between dentin and cementum is marked (white arrows). In (**C**), trichrome staining reveals non-calcified periodontal tissue (pdt) compatible with the periodontal ligament, along with other hard tissues (cementum, dentin, and bone). In (**C**,**D**), cementum is observed over dentin and bone tissue (white arrows).

A total of twelve implants diagnosed with peri-implantitis were extracted as part of a descriptive study on peri-implant diseases conducted at the Implant Clinic, Faculty of Dentistry, Universidad de La Frontera (UFRO), Temuco, Chile, and the Peri-implant Clinic, Faculty of Dentistry, Universidad de Concepción (UdeC), Concepción, Chile. The clinical protocol was approved by the Ethics Committee of Universidad de La Frontera (024-2018). The implants were extracted using a trephine of sufficient diameter to ensure a margin of at least 0.5 mm between the implant surface and the internal surface of the trephine. The samples were fixed in 4% buffered formalin for 72 h. For histological analysis, each complete specimen (implant plus surrounding tissue) was processed for plastic embedding. Subsequently, the samples were sectioned along the midline of the implant, dividing them into two segments. One segment was abraded, polished, and prepared for backscattered electron microscopy (BS-SEM) analysis. The other segment was used for histological analysis with trichrome staining, as described in the literature [3]. During analysis, one sample revealed that the implant made contact with the intraosseous remnant of a tooth root. (Figure 1). BS-SEM analysis revealed that bone tissue utilized the implant surface and dentin as scaffolds for regeneration (Figure 1A). Cementum was observed on the surfaces of the dentin and bone tissue (Figure 1C,D indicated by arrows). On the underside of the implant, an amorphous hard tissue resembling both bone and cementum occupied the space between the implant and dental tissue (Figure 1E,F).

### 2.2. Findings in the Animal Model

**Figure 2:**
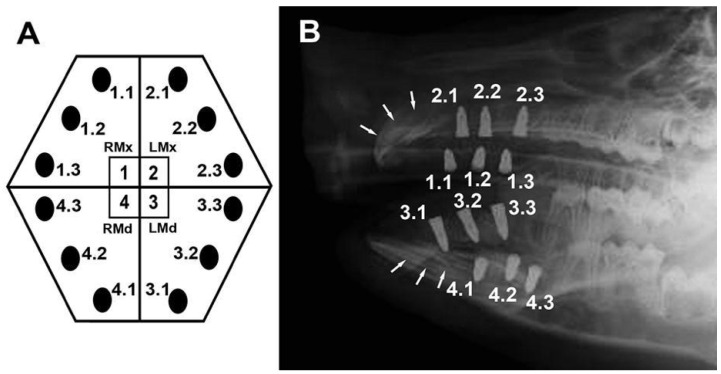
(**A**): Schematic illustration of implant distribution. Black circles indicate implant positions. RMx: Right maxilla; LMx: left maxilla; RMd: right mandible; LMd: left mandible. (**B**): Postoperative lateral radiograph showing implant distribution. White arrows indicate the position of the canines.

The biocompatibility study of dental implants with new surface treatments, conducted using two male pigs, was evaluated and approved by the Animal Experimentation Ethics Committee of the International University of Catalonia (06-2011). The animal protocols followed the principles of the 3Rs concerning the use of animal models in experimental research: Replacement, Reduction, and Refinement [4,5]. Furthermore, all procedures were carried out by specialized surgeons under veterinary supervision, and the care and management of the animals complied with the ethical standards outlined in the *Guide for the Care and Use of Laboratory Animals* [6]. A total of 24 implants (12 per animal) were placed in the maxillary and mandibular bones immediately following bilateral premolar extraction (Figure 2A). Postoperative lateral cephalic radiographs were taken to assess implant distribution (Figure 2B). Ninety days post-surgery, the animals were sacrificed, and the implants, along with surrounding tissues, were conditioned for embedding in light-curing resin for further analysis. The samples were then prepared for BS-SEM analysis and stained with toluidine blue for histological examination via light microscopy, as previously described [3].

**Figure 3:**
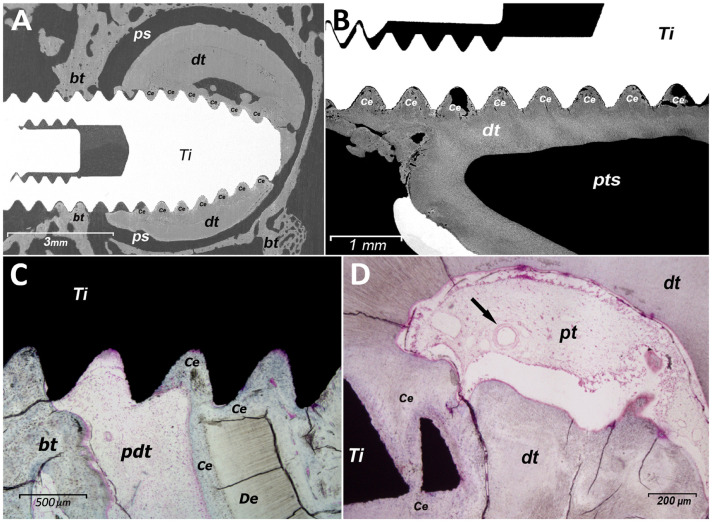
(**A**): SEM image of a titanium implant (Ti) 4.1 penetrating the canine tooth, surrounded by dental tissue (dt) and bone tissue (bt). (**B**): BS-SEM image showing dental tissue superficially eroded by implant 1.1, with its surface covered by cementum (Ce). (**C**): Histological image of implant 4.1 showing bone tissue and cementum formation on the surface of the implant threads (cementointegration), along with dentin (De). (**D**): Histological image of pulpal tissues (pt) in the periapical area of implant 4.1, stained with toluidine blue. The black arrow indicates a blood vessel. ps: Periodontal space; pdt: periodontal tissue; pts: pulpal tissue space.

Postoperative radiographs revealed that the implants placed anteriorly were situated near the canine teeth (implants 1.1, 2.1, 3.1, and 4.1 in Figure 2), which have long roots extending antero-posteriorly within both the maxillary and mandibular bones. Subsequently, during the sectioning process of samples embedded in plastic, implants 1.1 and 4.1 from specimen 1 were found to be in contact with dental tissues. Despite this, the animals exhibited normal behavior and feeding patterns, without any signs of discomfort during the observation period. Histological analysis revealed that implant 4.1 had penetrated the surface of the mandibular canine, while the right maxillary canine was superficially contacted by implant 1.1. In histological images obtained via BS-SEM (Figure 3A,B), tooth tissue formation was evident between the implant threads. Examinations of implant 4.1 and the surrounding tissue showed that bone, periodontal ligament, and tooth tissue (cementum) had adapted to the implant’s surface (Figure 3C). In both implants, new cementum and bone were observed on their surfaces, originating from the adjacent tissue, indicating the simultaneous integration of the implant by both bone and tooth tissue. Furthermore, histological analyses of the mandibular canine revealed pulp tissue exhibiting characteristics of vitality (Figure 3D).

**Figure 4:**
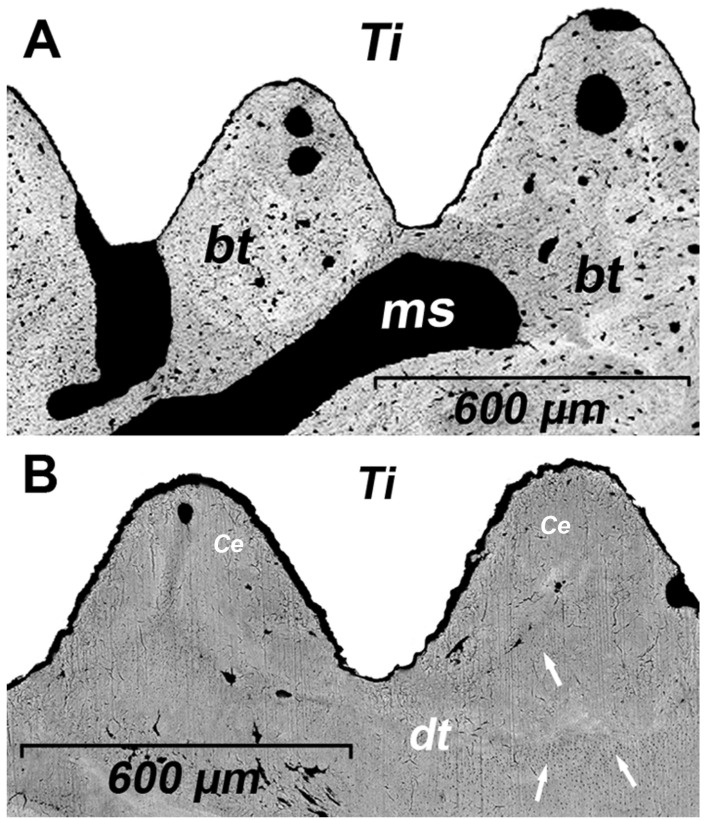
(**A**): BS-SEM image of threads from the osseointegrated dental implant 4.3 (specimen 1). (**B**): BS-SEM image illustrating dental tissue (dt) in contact with the implant’s surface (implant 4.1), consistent with acellular cementum, demonstrating the cementointegration of the implant. Dentin is identified via the presence of dentinal tubules (white arrows). Cementum (Ce) is observed forming between the dentin and the implant surface. bt: Bone tissue; ms: marrow space; Ti: titanium dental implants.

The BS-SEM images revealed differences in implant surface integration via osseous tissue (Figure 4A) and cementum (Figure 4B). The cementointegration process involved the use of dentin and cementum as a scaffold (Figure 3C) and direct integration with the implant’s surface. The morphology of the cementum predominantly corresponded to that of acellular cementum. Furthermore, cementum demonstrated a greater capacity than bone for filling the spaces between the implant threads (Figure 3 and Figure 4).

## 3. Discussion

### 3.1. Experimental Implications on Dental and Periodontal Tissues Contacted by Dental Implants

In the animal findings described here, dental implants were integrated into periodontal tissues through the reparative activity of both soft tissue and cementoblasts following dental damage (Figure 2, Figure 3 and Figure 4). This phenomenon demonstrates a pattern of cementum behavior similar to that of bone tissue on the implant surface, which allows it to be described as “cementointegration”, a concept rarely addressed in the literature. Several studies have reported similar findings. Buser et al. [7,8] demonstrated the formation of periodontal tissue around dental implants, with cementum formation on cylindrical titanium implants in contact with retained dental roots, using a monkey model. Warrer et al. [9] obtained comparable results using a similar experimental design and animal model, but with a self-tapping screw-type implant system. In contrast, Gray and Vernino [10], in an analogous experiment with baboons, did not observe periodontal ligament formation, but did find cementum on the implant’s surface. New cementum formation was also reported by Hürzeler et al. [11], who, using an animal model, inserted an implant into a dental socket with a retained root fragment in a procedure known as the “socket-shield technique”, designed to prevent alveolar bone resorption. The formation of new cementum on both dental tissue (dentin) and implant surfaces is a consistent observation in experimental and incidental findings in the studies mentioned above.

Understanding how cementum initially contacts and continues to cover the implant surface and identifying which implant surface treatments or microenvironments best promote this phenomenon remain critical challenges. Urabe et al. [12] demonstrated that while the bioactivity of the implant material did not affect the migration of periodontium-derived cells, it significantly influenced cell differentiation. Hürzeler et al. [11] proposed a potential relationship between the use of enamel matrix derivatives and cementum formation on the implant surface.

The quantity of cementum may also be a critical factor. As illustrated in Figure 3 and Figure 4, cementum demonstrated a greater capacity than bone to fill the space between the implant threads. These results are comparable to those observed in a study carried out by Hürzeler et al. [11].

The findings described here, along with the studies mentioned previously, demonstrate the response of vital cementum (characterized by the presence of active cementoblasts) in contact with dental implants. However, the effect of implants on non-vital cementum has not been as widely documented. In this context, the closest evidence was presented by Davarpanah et al. [13], who placed an implant in contact with an ankylosed dental root, achieving stability and realizing the integration of the implant with both dental and bone tissues. Similarly to the use of dentin as a biomaterial for the osteoconduction of new bone [14,15], the acellular cementum present in the implant bed may serve as a natural scaffold for bone regeneration

Although the characteristics of the implant’s surface (such as material, porosity, and treatment) can influence both osseoconductivity and cementoconductivity, these phenomena also depend on specific molecular and cellular interactions. In a study by Parlar et al. [16], which investigated the formation of periodontal tissue on titanium surfaces, it was observed that the presence of periodontal tissue inhibited osseointegration. In this context, it is known that cementoblasts from the periodontal ligament express cementin 1 protein (CEMP1), which reduces the expression of Runx2 and osteocalcin genes [17], both of which are essential for osteoblast differentiation. Our findings in the animal model demonstrated that bone, periodontal ligament, and cementum healed and covered the implant surface, maintaining their respective positions even when the implant traversed the periodontal and dental structures (Figure 3C). In the human case, bone formation was observed utilizing both the implant surface and dentin as scaffolds, with cementum forming on dentin and bone tissue, although no direct contact was observed between cementum and the implant (Figure 4). Despite this, the amorphous hard tissue observed adjacent to the implant surface (aht, Figure 1) may represent tissue formation resulting from the complex interaction between bone and cementum. As a precedent, Rinaldi and Arana-Chavez [18] and Hürzeler et al. [11] described a dense amorphous material containing collagen fibrils, which formed a cementum-like layer over an implant in contact with the dental root. Further studies on this amorphous tissue are likely necessary to clarify the bone–cementum interaction during the integration of dental implants with bone and periodontal tissue. On the other hand, in experimental implant insertions that caused superficial damage to healthy tooth roots (as shown in Figure 3B), bone and periodontal tissue regeneration was observed, with a cementum layer formed on the implant surface but without significant alterations in tissue positions; both the tooth and implant remained healthy throughout the experimental period [18,19]. Similar results were obtained in experiments without traumatic contact between periodontal tissues and implant surfaces. Jahangiri et al. [20] demonstrated that implant–tooth contact was achieved by applying controlled orthodontic force to move the tooth toward the implant-induced cementum growth on the implant’s surface, apparently through the transfer of cellular elements from the periodontal ligament.

Another experimental implication of the observations regarding implant–tooth contact relates to the formation of periodontal tissues on dental implants through a combination of in vitro and in vivo experimentation. For example, Choi [21] demonstrated that cultured periodontal ligament cells could induce the formation of cementum and ligament when placed on the implant surface and subsequently inserted into the mandibular bone of dogs. In another example, Marei et al. [22] placed implants combined with polymer scaffolds enriched with undifferentiated mesenchymal stem cells into the mandibular bone of goats, demonstrating that these cells were able to differentiate and promote the formation of cementum, bone, and periodontal ligament.

### 3.2. Clinical Implications of Contact Between Dental Tissues and Implants

There has been a growing interest in clinical approaches involving implant–tooth contact for various objectives (Table 1). Hürzeler et al. [11], followed by Bäumer et al. [23], implemented the socket-shield technique, which involves retaining a portion of the root in areas where the alveolar bone is thin during implant bed preparation, with the goal of preventing bone resorption. This method was initially evaluated in animal models, where it was reported that cementum-like tissue formed on the implant surface and that the gingival architecture surrounding the implant remained well preserved after six months. Additionally, there is evidence supporting an innovative approach for implant placement in bone sites with impacted (unerupted) teeth, where extraction is avoided to reduce the risk of bone loss (Table 1). However, a series of six cases (a total of eight implants) presented by Langer et al. [24] documented the failure of three implants inserted in anatomical regions with retained root remnants. Similarly, Guarnieri et al. [25] described the failure of a dental implant placed in contact with a dental root due to the development of peri-implant disease one year after insertion. Furthermore, evidence suggests that the devitalization of teeth caused by an implant passing through the root of erupted healthy teeth ultimately led to implant extraction [26,27,28,29]. Consequently, clinicians must consider varying tissue responses when an implant contacts dental tissue from impacted or unerupted teeth, erupted teeth, or retained root remnants.

Managing trauma and inflammation may optimize cementum growth on the implant surface in contact with dental tissue. The literature suggests that a proinflammatory stimulus (such as cytokines combined with compressive forces) can reduce the expression of bone sialoprotein and CEMP1 [30], both of which are essential for cementum formation [17,31]. Bone sialoprotein is a component of the extracellular matrix in mineralized tissues, and it plays a critical role in hydroxyapatite precipitation [32]. This protein is found in both cellular and acellular cementum structures [33], and its absence can result in significant defects in acellular cementum formation and periodontal attachment [31]. Moreover, Wang et al. [34] demonstrated through in vitro and in vivo studies that proinflammatory cytokines can impair cementum regeneration. Optimizing the conditions for cementum formation may even encourage more favorable coverage of the implant surface by cementum compared to bone. Studies on graft materials used for periodontal apparatus regeneration have shown that cementum covers the tooth surface faster and earlier than bone tissue [35,36]. This finding aligns with the observations in Figure 3, where cementum is seen to cover the implant surface more extensively than bone.

The manner in which the implant contacts the tooth and the status of the tooth in the oral cavity (unerupted, erupted, root remnant, etc.) appear to be important factors to consider in analyzing this subject. Table 1 schematically presents findings from the literature regarding the outcomes of titanium implants in contact with dental tissue in both animal and human models, taking into account the previously described variables.

**Table 1:** Literature evidence from experimental and clinical reports on titanium implants in contact with dental tissue (including our findings).

Dental Implant Contacting Dental or Periodontal Tissues	Evidence in the Literature Author (Year)	Human/Animal Evidence	Clinical Observations, Tissues Reactions, and Histological Evidence
Implant passing through the dental or periodontal tissue from root of erupted, functional teeth.	Sussman (1998 a y b) [26,27]	Human	One case: Implant passing through the root of an erupted mature tooth, causing a periapical lesion. Endodontic treatment and implant extraction were indicated. No histological evidence is available.
	Margelos and Verdelis (1995) [29]	Human	Three cases: Implant apparently passing through the periodontal tissue in the apical area caused irreversible pulpal damage. Endodontic treatment and implant extraction were indicated. No histological evidence is available.
	Our findings, Figure 2A,C,D and Figure 4B	Animal	One implant passing through the root of an erupted tooth was integrated with both dental (cementum) and bone tissue.
Implant placed in bone sites with impacted teeth or supernumerary (passing through unerupted teeth)	Ouni and Mansour (2023) [37]	Human	One case: Two implants were placed through retained teeth in the mandibular bone of a patient with amelogenesis imperfecta. One implant evolved successfully (clinically stable after 36 months), while the other required replacement due to failure. No histological evidence is available.
	Brinkmann et al. (2020) [38]	Human	One case: Two implants were successfully placed through unerupted teeth in a patient with multiple impacted teeth. The implants remained clinically stable after 24 months. No histological evidence is available.
	Davarpanah et al. (2012, 2015) [13,39]	Human	Ten cases: A total of 15 implants were successfully placed through unerupted teeth. All implants remained clinically stable, with follow-up periods ranging from 1 to 8 years. No histological evidence is available (some cases were previously reported)
	Szmukler-Moncler et al. (2014) [40]	Human	One case: An implant was successfully placed through an unerupted tooth. The implant remained clinically stable after 18 months. No histological evidence is available.
	Kaplansky and Kurtzman (2024) [41]	Human	One case: An implant was placed in the maxillary anterior region, passing through supernumerary teeth. The implant remained clinically stable after 3 years and 8 months. No histological evidence is available.
Implant passing through a dental root remnant (or retained root)	Our finding, Figure 1	Human	One case: Implant passing through a retained root. Implant was extracted due to peri-implant disease. Histological analysis showed new bone tissue and amorphous hard tissue (likely a mixture of cementum and bone tissue) in contact with the implant surface.
	Langer et al. (2015) [24]	Human	Six cases: Eight implants in contact with undetected root fragments. Osseointegration issues were observed in all implants, but only three were extracted. The remaining implants were surgically treated to remove the root remnant. Histological analysis of one sample showed acellular cementum on root fragments, with no histological evidence regarding the implant.
	Szmukler-Moncler et al. (2015) [42]	Human	Six cases: A total of seven implants were successfully placed through root remnants. All implants remained clinically stable, with follow-up ranging from 20 months to 9 years. No histological evidence is available.
	Baümer et al. (2013) [23]	Human	One case: Implant successfully placed using the socket-shield technique. Implant remained clinically stable after 6 months. No histological evidence is available.
	Davarpanah et al. (2012) [13]	Human	Two cases: Implants successfully placed through dental roots. One case involved an ankylosed root, and the other an endodontically treated root. Both implants remained clinically stable (after 32 and 20 months, respectively). No histological evidence is available.
	Hürzeler et al. (2010) [11]	Human	One case: Implant placed successfully using the socket-shield technique. Implant remained clinically stable after 6 months. No histological evidence is available.
	Davarpanah et al. (2009) [43]	Human	Five cases: Implants successfully placed through ankylosed dental roots. All implants remained clinically stable (follow-up ranged from 12 to 42 months). No histological evidence is available.
	Guarnieri et al. (2002) [25]	Human	One case: Implant was removed after one year due to peri-implant disease. Histological analysis showed formation of cellular cementum on the implant surface.
	Baümer et al. (2013) [23]	Animal	Twelve implants placed successfully using the socket-shield technique. Healthy periodontal tissues and new bone observed between implant and dentin. Cementum was not observed.
	Hürzeler et al. (2010) [11]	Animal	One implant successfully placed with the socket-shield technique, showing integration in dental and bone tissue. Areas between implant threads near the root fragment were partially filled with cellular cementum, amorphous mineralized tissue, and connective tissue.
	Gray and Vernino (2004) [10]	Animal	Ten implants successfully placed through remnant root tips. Implant integration in dental and bone tissue with cementoconduction on the implant surface was observed.
	Warrer et al. (1993) [9]	Animal	Eight implants successfully placed through remnant roots. Implant integration in dental and bone tissue with cementoconduction on the implant surface was observed. Also, formation of periodontal ligament was described.
	Buser et al. (1990a) [7]	Animal	Six implants successfully placed through remnant roots. Implant integration in dental and bone tissue with cementoconduction on the implant surface was observed. Also, formation of periodontal ligament was described.
Implant traumatically contacts the root surface of the tooth.	Our finding, Figure 3B	Animal	One implant contacting the root surface from erupted teeth was integrated by dental (cementum) and bone tissue.
	Urabe (2000) [12]	Animal	Twelve implants placed in contact with dental and periodontal tissue experimentally. Cementum-like tissue and periodontal ligament were observed mainly on the implant surface covered with hydroxyapatite.
	Asscherickx K et al. (2005) [19]	Animal	Three roots contacted by mini-implants for orthodontics, then removed. Histological examination of these roots demonstrated almost complete repair of the periodontal structure
	Rinaldi and Arana-Chávez (2010) [18]	Animal	Twenty-four mini-implants placed in contact with dental and periodontal tissue experimentally. The alveolar bone and periodontal ligament reorganized around the implant, forming a thin cementum-like layer over time at contact points with the periodontal ligament.

### 3.3. Applications and Challenges Arising from Evidence of Tooth–Implant Contact

Titanium dental implants achieve direct integration with bone but lack certain essential functional structures found in natural teeth, such as cementum and the periodontal ligament. These structures collectively function to absorb and buffer masticatory forces, which could provide the implant with properties that manage chewing stress, similarly to that endured by a natural tooth. The literature has introduced concepts such as the “functional periodontal ligament tissue formation on titanium implants” [44], the “bio-hybrid implant” [45,46] and the “functional implant” [47], developed through dental tissue engineering, which describe an implant that interacts with the bone through newly formed periodontal-like tissue. Washio et al. [45] demonstrated the creation of periodontal-like tissue around titanium implants under certain conditions using the “Cell Sheet Engineering Technology”, transforming in tissue engineering what had been observed in different findings and clinical experiences (Table 1). Another concept associated with the creation of a peri-implant biological apparatus similar to dental periodontal tissue is that of “Ligaplant” [48,49]. Bio-hybrid implants and ligaplants, developed through tissue engineering, enable the construction of an implant-to-bone adhesion system similar to the natural tooth–bone union, incorporating cellular elements relevant for maintaining its physiology. However, achieving a more extensive coverage of the implant surface with cementum, similarly to that observed across the entire natural dental root, remains a considerable challenge. To date, it has been demonstrated that cementum has a greater capacity than bone to fill the space between the implant threads; nevertheless, it is mainly an acellular tissue and lacks the natural remodeling properties found in osseous tissue, for example. Therefore, at present, the coverage of implant surfaces with cementum is only relevant if it forms part of a more complex periodontal structure, including the periodontal ligament with cellular elements capable of participating in the remodeling and repair of the cementum formed on the implant surface [50]. Another significant challenge is realizing a sufficient functional proprioceptive response from the neo-structure to provide the implant with protection similar to that of a tooth with natural periodontal tissue.

Regarding advances in the science of biomaterials applied to new generations of dental implants, various options have been described that could contribute to the development of periodontal-like tissue around implants, such as the use of implant surface treatments via electrospinning, which enables the implant’s surface to be loaded with biopolymers enriched with biomolecules [51] that could stimulate and protect tissue formation, or the use of nanoenzymes that promote tissue regeneration [52]. These surface treatments are independent of the material used to create the implant; however, it is important to consider that there is limited information on the behavior of periodontal tissue, particularly cementum, with respect to implants made from materials other than titanium.

### 3.4. Limitations

Although there is some clinical evidence regarding the viable placement of dental implants in contact with retained teeth or root remnants, establishing parameters for success or failure in this type of intervention is complex. The successful cases presented in the literature exhibit significant variations in terms of implant characteristics, type of rehabilitation applied to the implant, health status of the bone and dental structure where the implant was inserted, and patient history (age, general health, facial biotype, etc.). Consequently, the level of evidence is low, and the procedure cannot be standardized for consistent replication. Nevertheless, successful cases suggest that this treatment option could be considered a viable alternative subject to the patient’s informed consent.

Regarding the experimental protocols analyzed, they generally converge on the concept of producing periodontal-like tissue around titanium implants. However, there is insufficient evidence to predict the long-term subsistence of the newly formed tissue under masticatory forces or its functional proprioceptive capacity. Moreover, it has been reported that these therapeutic alternatives have high costs and low predictability [48]. Finally, the use of titanium dental implants within this novel framework of interaction with human tissue raises new questions regarding biosafety, given the concerns expressed by some authors about the potential toxicity of the metal [50].

## 4. Conclusions

The findings described here, along with the literature review, indicate that titanium dental implants possess cementoconductive capacity. This property can be modified by altering the morphological or bioactive characteristics of the surface. Furthermore, it has been demonstrated that dental implants can integrate with both bone and dental tissue simultaneously when placed in contact with both structures under controlled conditions. In this context, clinical applications represent an intriguing treatment alternative that includes preparing an implant bed that traverses both the bone and dental tissue of impacted teeth or retained roots. Clinicians must consider that long-term evidence regarding the durability of this treatment alternative is limited. Additionally, the potential for periodontal tissue formation around dental implants has served as a foundation for tissue engineering studies aimed at achieving implant insertion in bone via periodontal-like tissue. The experimental success of tissue engineering in forming periodontal-like tissue for dental implant insertions requires controlled studies to assess its long-term utility under physiological conditions.

## Data Availability

The original contributions presented in the study are included in the article, further inquiries can be directed to the corresponding author.
